# Temperature prediction of solar greenhouse based on NARX regression neural network

**DOI:** 10.1038/s41598-022-24072-1

**Published:** 2023-01-28

**Authors:** Maosheng Gao, Qingli Wu, Jianke Li, Bailing Wang, Zhongyu Zhou, Chunming Liu, Dong Wang

**Affiliations:** 1 Meteorological Administration of Yangling City, Yangling, 712100 China; 2 Yangling High-Tech Agricultural Meteorological Technology Combined Research Center, Yangling, 712100 China; 3 Shaanxi Meteorological Cadre Training College, Xi’an, 710016 China; 4 College of Mechanical and Electronic Engineering, Northwest Agriculture and Forestry University, Yangling, 712100 China

**Keywords:** Plant sciences, Photosynthesis

## Abstract

Temperature has an important influence on plant growth and development. In protected agriculture production, accurate prediction of temperature environment is of great significance. However, due to the time series, nonlinear and multi coupling characteristics of temperature, it is difficult to achieve accurate prediction. We proposed a method for building a solar greenhouse temperature prediction model based on a timeseries analysis, that considers the time series characteristics and dynamic temperature changes in the greenhouse system. The method would predict the temperature of greenhouse, and provide reference for the temperature change law in protected agriculture. A parameter analysis was performed on the nonlinear autoregressive exogenous (NARX) neural network, and a solar greenhouse temperature time series prediction model was established using the NARX regression neural network. The results showed that the proposed model depicted a maximum absolute error of 0.67 °C, and model correlation coefficient of 0.9996. Compared with the wavelet and BP neural networks, the NARX regression neural network accurately predicted and significantly outperformed in the solar greenhouse temperature prediction model. Moreover, the prediction model can accurately predict temperature trends within the solar greenhouse and is pivotal to obtaining precise control of solar greenhouse temperature.

## Introduction

Solar greenhouses are designed to insulate heat and increase production^1,2^. In recent years, the area of greenhouses in China has continued to expand^3,4^. As of 2020, greenhouses covered 340 hectares, 90% of which are solar greenhouses located in northern China^5^. Solar green houses are operated by simple, manual processes. In winter, the manager will close the greenhouse based on experience to ensure the indoor temperature^6^. However, because these temperature control decisions are made in the absence of scientific assessment, they are often erroneous or tardy, which leads to delays in shed opening time, negatively impacts the greenhouse’s sunlight exposure time in winter, and contributes to a decline ineffective crop photosynthesis^7,8^. Therefore, to achieve effective greenhouse temperature regulation, it is particularly important to predict the greenhouse temperature in advance^9^.

Solar greenhouses are nonlinear, large inertia, strong coupling, and time-varying complex systems^10^. As such, implemented control measures and changes in external climate will impact the greenhouse environment and thus, facilitate changes in the solar greenhouse crops’ physiological characteristics^11^. The modeling methods of greenhouse temperature can be divided into mechanism models based on energy balance and non-mechanism models based on data drive. The mechanism model has the advantages of strong interpretability, but it is difficult to apply to the actual agricultural production due to its low accuracy, poor versatility and difficulties in obtaining parameters. With the development of sensor technology, data acquisition becomes easier and easier. Data driven model is widely used in agriculture because of its high precision and strong generalization ability. Holthuijzen^12^ has used five statistical methods to predict temperature. Although statistical methods can predict time series data, their ability to deal with nonlinear problems is limited, and their fitting accuracy is generally low.

To circumvent this issue, many scholars use intelligent algorithms to solve the challenges associated with greenhouse modeling. Examples include but are not limited to: fuzzy control, neural network, genetic, and nonlinear regression algorithms^13–16^. Among them, the application of neural networks in greenhouse modeling is particularly prominent^17^, as modeling process difficulties are greatly reduced due to the neural networks’ black box nature^18^. Ferreira et al.^19^ used radial basis function neural network to predict the greenhouse temperature. The parameters of the model were optimized by Levenberg–Marquardt method. However, the model belongs to static neural network and the temperature time series change information is not considered. The long-term prediction accuracy is low.

Considering the time series change characteristics of temperature, we designed a closed loop neural network Nonlinear Auto Regressive Model with Exogenic Inputs (NARX). Compared with the traditional neural network, this network takes the predicted temperature as the feedback input, which belongs to a dynamic model. The closed loop network can predict the temperature according to the previous temperature to improve the accuracy of the model. In order to train the model easily, the error back propagation algorithm was used to train the parameters. In researches of temperature prediction, NARX has dynamic time series characteristics and is convenient for training.

In this work, a dynamic neural network was employed to develop a model that enables solar greenhouse temperature series prediction with time series characteristics. Herein, the time series concept, which is based on traditional neural network modeling, is introduced; and a NARX recurrent neural network (NARXNN) prediction model with two primary external input influencing factors—light and temperature difference—is proposed. Results show that the prediction accuracy and network performance of a NARXNN prediction model based on time series analysis is significantly better than that of other algorithm models, which has practical significance for accurate regulation of greenhouse temperature.

## Materials and methods

### Testing platform structures

In this work, greenhouse temperature and temperature-related environmental data were experimentally obtained, from December 12–17, 2021. Testing was conducted at Northwest A&F University of Agriculture and Forestry Science and Technology in Wutun Town, Yanliang, Xi’an City (latitude 34° 35′ 11″ N, longitude 109° 08′ 54″ E). The experimental greenhouse was 46 m long and3.8 m high, with 1 m thickeast, west, and rear walls. Experimental apparatus consisted of a db130 air temperature and humidity sensor, with a measurement accuracy of ± 0.5 °C at ambient temperature (25 °C); and a DAVIS6450 solar radiation sensor, with a ± 5% measurement accuracy at full scale.

**Figure 1:**
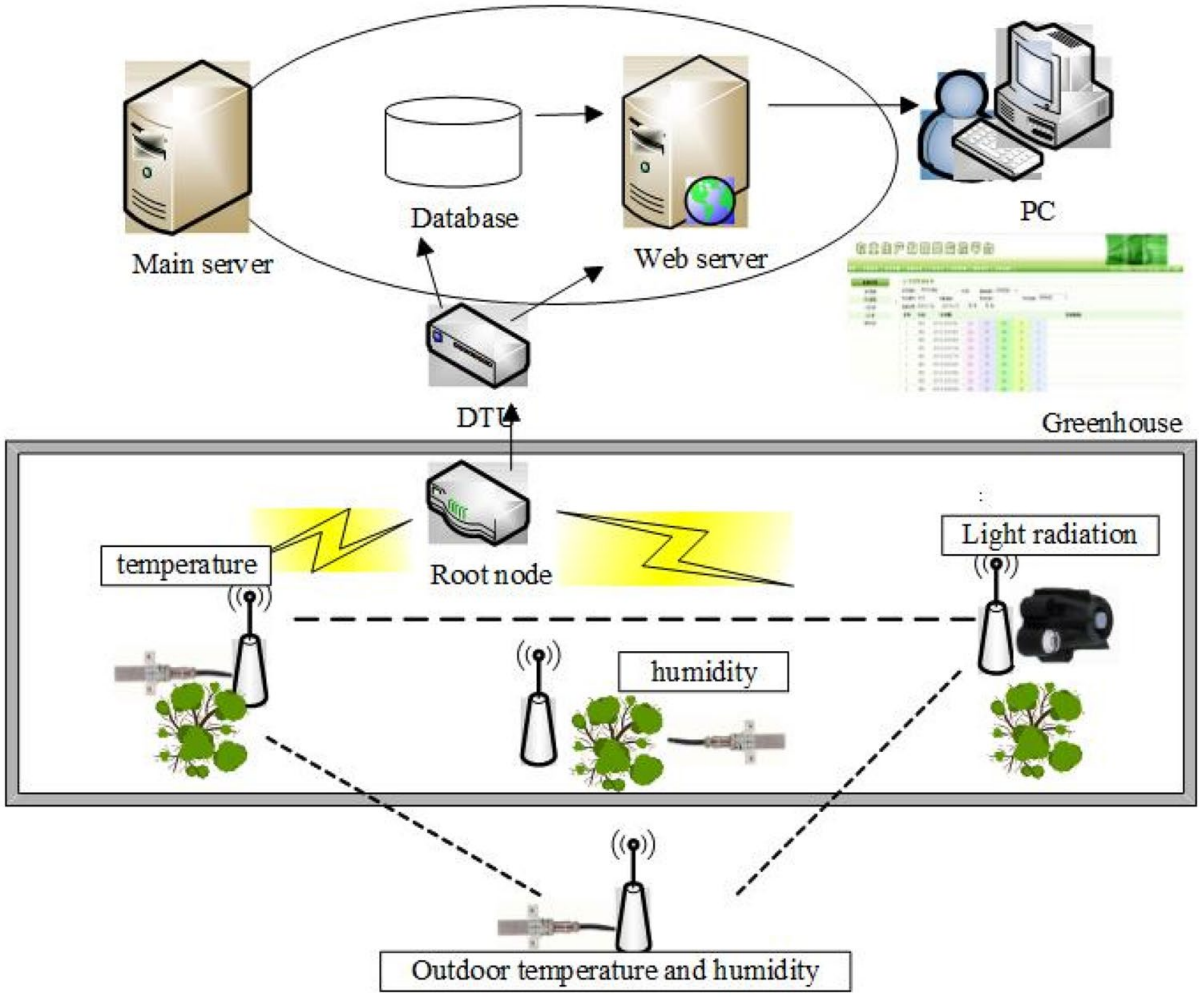
Experimental data acquisition system.

Using ZigBee wireless communication and general packet radio service (GPRS) technology, the monitoring node collected the greenhouse environmental factor information, and uploaded the data to the website through the root node and DTU. The test data acquisition system is shown in Fig. 1.

The monitoring node is composed of a power supply module, core processing module, sensor module, and debugging module. All the monitoring nodes are networked to collect and transmit the environmental data from each node—which consists of indoor/outdoor air temperature, humidity, and light radiation data that has been collected every 5 min. The root node is composed of the serial port, power, and core processing modules. Using ZigBee wireless sensor network technology, the latter receives the data collected by each monitoring node and sends it to the DTUs through the RS232 serial port. The central server then uses GPRS technology to have the DTU upload the environmental factor information to the agricultural production Internet of Things monitoring platform. Users can then access the platform online to obtain test data.

#### Experiment design

**Figure 2:**
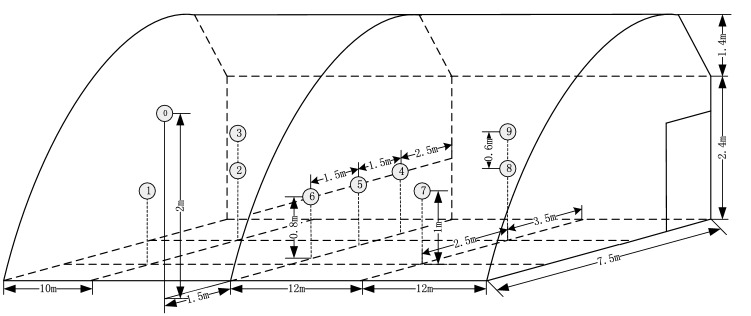
Schematic diagram of the solar greenhouse architecture and sensor distribution.

The greenhouse remained vacant during the experiment to avoid the influence of crops on indoor temperature changes. The temperature detection values differed as a function of height and distance from the rear wall, so the placement position of each sensor was determined as shown in Fig. 2. Nodes 1, 2, 7, and 8 were1m equidistant from the ground; Nodes 2, 3, 8, and 9 were3.5 m equidistant from the back wall; Nodes 4, 5, and 6 were0.8 m equidistant from the ground within the same vertical plane; and Nodes 1, 2, and 3 were symmetrical with nodes 7, 8, and 9 relative to the vertical planes of nodes 4, 5, and 6. In addition, Nodes 1-9 were outfitted with air temperature and humidity sensors, while nodes 4, 5, and 6 were outfitted with light radiation sensors. Node 0 was located outdoors, at a minimum distance of 1.5 m from the greenhouse, and a height of 2 m. This node was also outfitted with an air temperature/humidity sensor and light radiation sensor.

The test was conducted during clear weather conditions, such that the low temperatures and absence of light associated with actual extreme weather could be simulated. The greenhouse remained in an opened state from Dec.24th–25th, during which time the greenhouse quilt remained open at night. The greenhouse was closed before sunset on the evening of the 25th and open after sunrise on the morning of the 27th, during which time the quilt remained closed during the day. The shed was then closed again at sunset on the evening of the 28th. The specific time associated with opening and closing the shed in the morning and evening was managed by the greenhouse manager and performed in conjunction with the greenhouses of other crops. The five-day data collection time spanned from 00:00 on January 24, 2018 to 00:00 on January 30, 2018.After the data was obtained, the average temperature value of each node in the greenhouse was calculated and taken as the indoor temperature data, while the indoor/outdoor temperature difference was calculated and used for indoor/outdoor temperature difference data. After the outliers in the test data were excluded, the sample set consisted of 1440 sets of environmental factors.

#### Pretreatment of test data

Because temperature, temperature difference, and radiation intensity are characterized using different dimensions, the data could not be directly compared. As such, if the NARXNN model was established using the raw data, the model accuracy would be negatively impacted. Thus, the sample set was first normalized using the normalization mapping formula in Formula (1):

1 $$ y = \frac{{x - x_{min} }}{{x_{max} - x_{min} }} $$

where x, y ∈ R^n^; x_min_ = min (x); x_max_ = max(x), x is the data to be normalized, and y is the normalized data. Using this formula, the original data was normalized to the range of [0, 1].

To reserve sufficient opening action time and temperature response time, andto ensure prediction accuracy, 20 min was selected as the prediction time. Equal spacing started from the first data point in the first 1440 sets of data. Next, 361 sets of data i.e., every 4th set, was selected to comprise the new sample set. Of the reduced sample set, the first 300 data sets were used as the training set and the last 61 sets as the prediction set.

#### Greenhouse temperature prediction model based on NARXNN

In this work, a NARXNN time series prediction model with external input was used to establish a corresponding greenhouse temperature prediction model with the aim of resolving the temperature prediction challenges associated with solar greenhouse. The model was developed using static neurons and network feedback, and combined the linear autoregression (ARX) model’s nonlinear processing with a neural network, giving it strong nonlinear identification ability.

#### NARXNN based greenhouse temperature prediction model methodology

**Figure 3:**
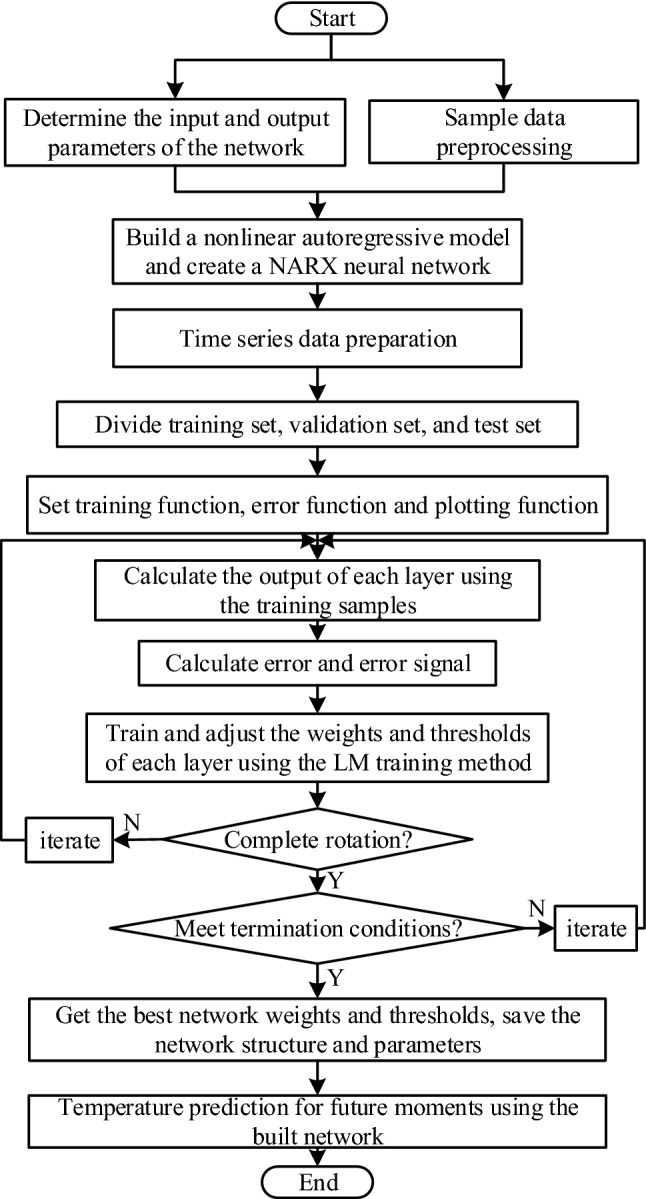
Specific flowchart of the NARXNN algorithm.

Using the sample set described above, a NARX regression neural network prediction model was developed. The greenhouse’s historical and current light radiation time and inside/outside temperature difference were used as inputs, while the greenhouse’s future temperature at a given time served as the output. A flowchart of the NARXNN algorithm is shown in Fig. 3. First, the appropriate prediction model structure was selected based on the network’s input and output characteristics, a nonlinear autoregressive neural network model with external input was constructed, and the network mode was simultaneously established. Next, the time series data was pre-prepared, and the 361 training sets were divided into new training sets, verification sets, and test sets in a 70:15:15ratio. Then, the network parameters were determined and the algorithm was taught to achieve network training. Finally, predictions were made using the trained network.

#### Network pattern selection

**Figure 4:**
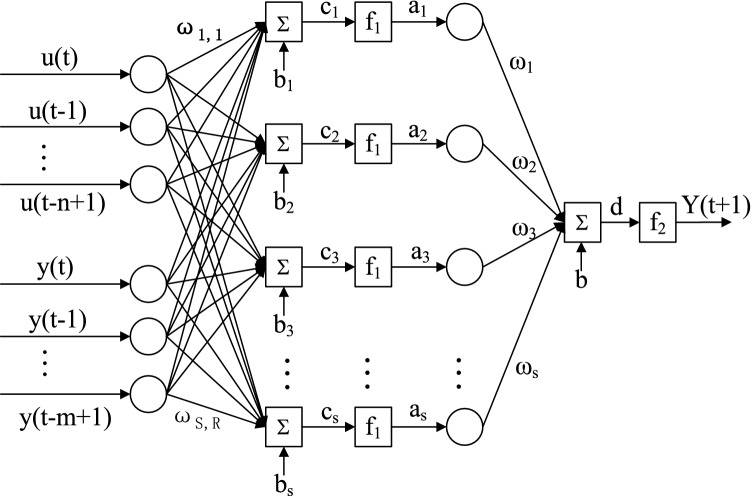
Series parallel neural network model.

The standard NARXNN operates in Parallel neural network mode^20^. Given the time series continuity, the prediction output is fed back as one-dimensional input of the next moment to add the historical series influence on the future moment’s predicted value. In practice, temperature data is collected and stored in real time, and the indoor temperature of the current and historical moments are known when making predictions. Therefore, a static network was built with the actual historical temperature value as one of the dimensional inputs. This resulted in a Series–Parallel neural network mode that turns the NARXNN into a pure forward neural network. Compared with the traditional BP neural network, the network structure adds a delay order, so the static neural network modeling function can be directly used, which reduces modeling difficulty and improves the network’s prediction accuracy to a certain extent. The network pattern is shown in Fig. 4.

In Fig. 4, n and m represents the system’s input and output delay orders, respectively; R = n + m and indicates the current network’s number of input nodes; ω_i,j_ is the connection weight between the *i*th neuron in the network's hidden layer and the *j*th element in the input vector; S is the number of hidden nodes; b_i_ is the *i*th neuron’s bias value in the network’s hidden layer; c_i_ is the net input of the *i*th neuron in the hidden layer; f_1_isthe transfer function of a crypto layer neuron; a_i_ is the output of the *i*th neuron in the hidden layer; ω_i_ is the *i*th connection weight between the network output layer neurons and the hidden layer neurons; b is the neurons’ bias value in the network’s output layer; d is the net input of neurons in the output layer; f_2_isthe transfer function of neurons at the network’s output layer; and Y(t + 1) is the network’s predicted output.

If the current moment is t, the input–output relationship between the model and the t + 1 moment temperature value prediction is as follows^21^:

2 $$ {\text{Yt}} + 1 = \mathop \sum \limits_{i = 1}^{s} \omega_{i} f_{1} \left[ {c_{i} \left( t \right)} \right] + b $$

The vector form is shown in Formula (3):

3 $$ c_{i} \left( t \right)\mathop \sum \limits_{j = 1}^{n} \omega_{i.j} u\left( {t - j + 1} \right) + { }\mathop \sum \limits_{j = 1}^{m} \omega_{i,j + n} y\left( {t - j + 1} \right) + b_{i} $$

Formula (2) and Formula (3) are combined to obtain:

4 $$ Y\left( {t + 1} \right) = f\left( {u\left( t \right),u\left( {t - 1} \right), \ldots ,u\left( {t - n{ } + 1} \right),y\left( t \right),y\left( {t - { }1} \right), \ldots ,y\left( {t - m1} \right)} \right) $$

where u(t) is the temperature influencing factor term at the current moment, u(t-n + 1) is the temperature influencing factor term at the moment of t − 1 + 1, y(t) is the indoor temperature value at the current moment, y(t-m + 1) is the indoor temperature value at the t-m + 1 moment, and Y(t + 1) is the predicted indoor temperature at the next moment.

#### NARX regression neural network design

The selected input and output delay orders were n = 3 and m = 3—i.e., there were six input layer nodes.

**Figure 5:**
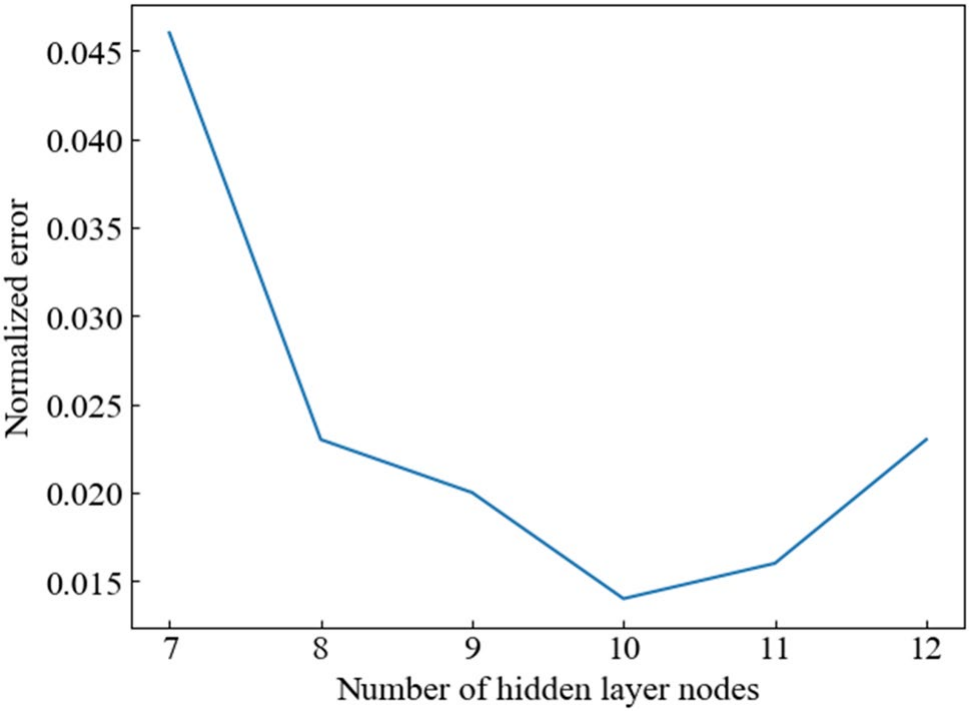
Performance function curve with the number of hidden layer nodes.

The number of neurons in the implicit layer is often based on experience, and in turn, has resulted in large network errors. To circumvent this issue, different numbers of neurons were tested for network training; and the number of neurons that produced the best training results was selected (Fig. 5).

The performance function is defined as the training result detection function and is used as the basis for determining whether the network training result is good or bad. As shown in Fig. 5, the NARXNN performance function value depicts an inverse relationship with the number of hidden layer nodes until the latter value reaches 10. At this point, the trend reverses and the two variables depict a positive correlation. While increasing the number of hidden layer nodes makes the network more accurate, it also results in a discordant fit, which affects the network’s performance. In this case, the performance value function is maximized when the number of nodes in the hidden layer is 10; thus, 10 was selected as the value for this variable. The prediction result was the temperature value of the next moment, and the number of nodes in the output layer was 1.

In summary, the number of network nodes was as follows: 6 input nodes, 10 hidden layer nodes, and 1 output node.

Neuronal activation functions are often divided into global functions—such as S-type functions, and local functions—such as RBF functions^22^. Since NARXNN is a global approximation network, its neuronal excitation function should be a global function. Therefore, the influence of different S-type transfer functions on network performance was investigated. Four models training step parameters were analyzed to determine the optimal transfer function combination. In addition, the training set’s root mean square error, deterministic coefficient, and training time were tested under different combinations of transfer functions. The results are shown in Table 1.

**Table 1:** The transfer function’s effect on the mode’s prediction accuracy.

Order number	Transfer function		Training step	RMS error	Coefficient of determination	Training time
	Implication level	Output level				
1	radbas	radbas	8	113.0576	0.7486	18.049
2	radbas	logsig	13	137.0880	0.1512	21.977
3	radbas	tansig	13	0.2326	0.9978	22.603
4	radbas	purelin	13	0.0224	0.9998	16.719
5	logsig	radbas	14	142.0744	0.06037	22.967
6	logsig	logsig	10	112.9237	0.7537	23.047
7	logsig	tansig	21	0.9222	0.991	20.620
8	logsig	purelin	7	0.0166	0.9998	18.409
9	tansig	radbas	7	116.1256	0.6604	22.727
10	tansig	logsig	25	112.8936	0.7565	24.207
11	tansig	tansig	19	0.0611	0.9995	23.625
12	tansig	purelin	15	0.0133	0.9999	19.9925
13	purelin	radbas	6	113.5387	0.7458	27.386
14	purelin	logsig	28	113.5672	0.7348	40.981
15	purelin	tansig	5	1.3032	0.9878	23.410
16	purelin	purelin	22	0.0172	0.9998	18.203

Table 1 shows that different combinations of transfer functions have an important impact on model training. The model rms error and deterministic coefficient obtained in the sequence numbers 3, 4, 7, 8, 11, 12, and16 depict superior performance. Among them, group 12 has the smallest root mean square error and the decision coefficient closest to 1, as well as a small training step and short training time. Thus 12 was selected as the optimal transfer function combination. Accordingly, the implicit layer’s transfer function was tansig, and the output layer’s transfer function was purelin.

There are numerous types of neural network learning algorithms. In this study, the error function and network computation time of the pseudo-Newtonian backpropagation algorithm, gradient descent backpropagation algorithm, Powell-Beale reset algorithm, gradient descent backpropagation algorithm for adaptive adjustment of learning rate, and Levenberg–Marquardt backpropagation algorithm were assessed and compared. The results are shown in Table 2.

**Table 2:** Evaluation index of different learning algorithms.

Predicted models	Training step	R^2^	RMSE	Operation time
trainlm	15	0.9999	0.0133	19.9925
trainbfg	29	0.9995	0.0487	20.271
traingd	0	27.3047	625.6171	14.239
traingda	170	0.9825	1.18318	22.806
traincgb	44	0.9976	0.2494	25.438

According to Table 2, the gradient descent backpropagation algorithm, which is known by the root mean square error value and the decision coefficient, is not suitable for the data characteristics and was therefore eliminated. Furthermore, the gradient descent backpropagation algorithm with the adaptive adjustment learning rate shows better adaptability, but still lags behind the remaining three algorithms. Comparing the LM backpropagation algorithm, BFGS learning algorithm, and Plwell-Beale reset algorithm, the LM backpropagation algorithm performed best—depicting a coefficient of determination = 0.9999, root mean square error = 0.0133, and requiringonly 15 trainingsteps. Considering the indicators that were compared, the LM backpropagation algorithm offered the optimal value, indicating that it can solve the actual problem faster and more accurately that the others. As such, the LM backpropagation algorithm was selected for this study.

In summary, the model used herein depicts the following parameters: network structure (3–10-1); hidden layer activation function (tagsig function); output layer activation function (purelin linear transfer function); training function (trainlm function); and the error performance function (mse function).

#### Model evaluation

In this work, the measured value and the predicted value were compared and used as the model evaluation benchmark. Moreover, the maximum absolute error, mean absolute error (MAE), root mean square error (MSE), decision coefficient, and calculation time were used as the specific evaluation indicators to determine the model’s advantages and disadvantages. The model’s feasibility was assessed by determining whether the indicators were within the acceptable range, while the model’s quality was evaluated by measuring the distance of each indicator from the best standard.

## Results and discussion

### Training effectiveness of the NARXNN prediction model

**Figure 6:**
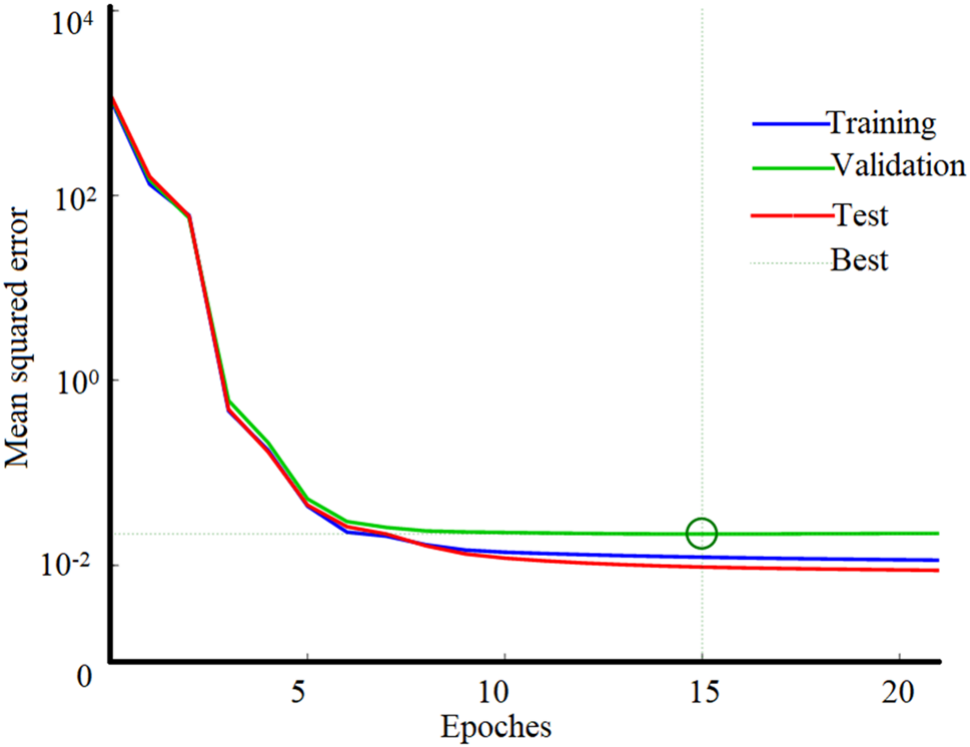
NARXNN training chart.

The already established NARXNN prediction model was used to train the study model, and the training effectiveness is shown in Fig. 6. Note that both the training set and test set reached the target error value in the 8th training. In contrast, the verification set reached the target error value after the 15th training, at which time the verification set error was 0.021865. Notably, the verification set error continued to rise, so the network required only 15 iterations to complete the training. These results indicate that the model prediction is characterized by a fast convergence speed and high accuracy.

### Feasibility of the NARXNN prediction model

**Figure 7:**
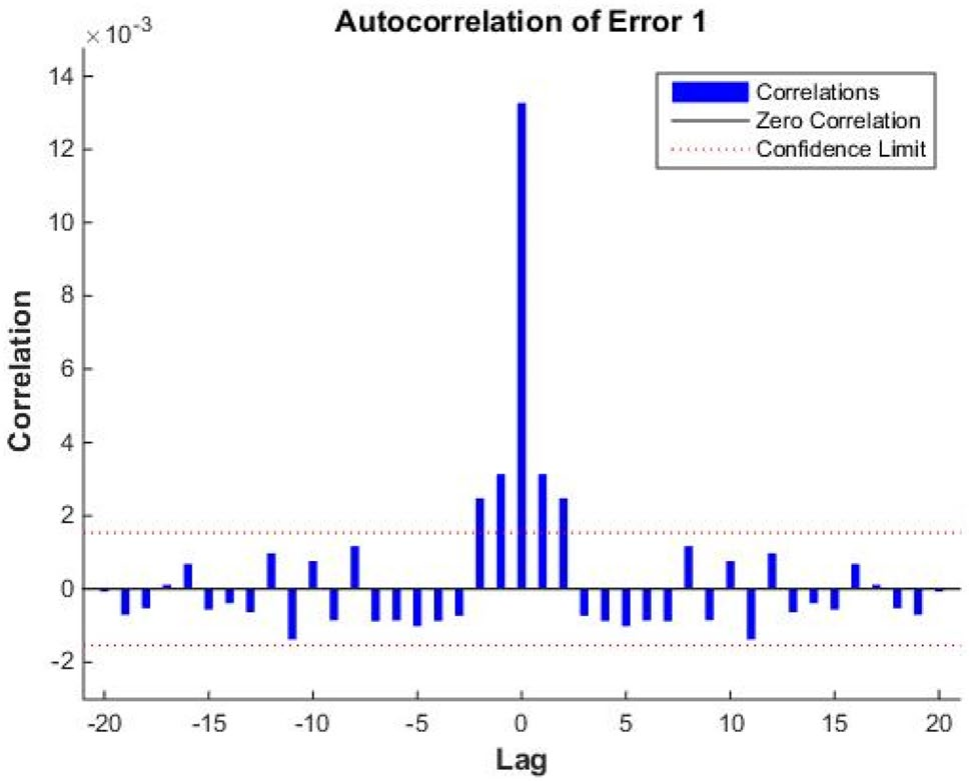
Error autocorrelation diagram.

**Figure 8:**
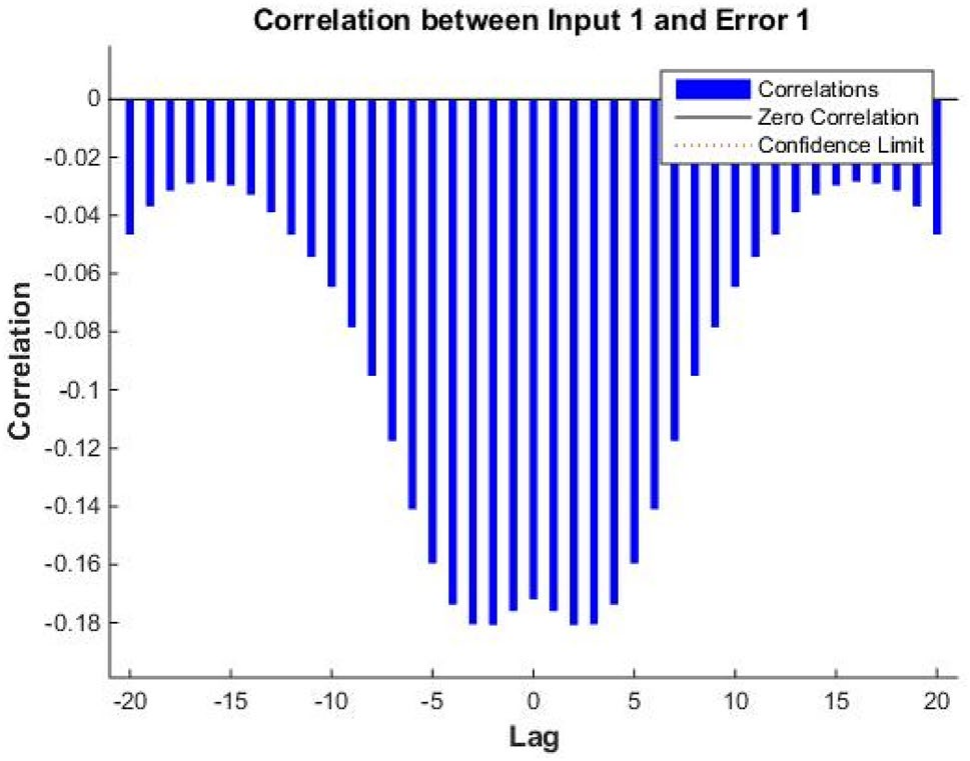
Input and output error correlation diagram.

A predictive model’s feasibility is usually determined by autocorrelation and cross-correlation tests. When the prediction error is obviously autocorrelated, and the input and error have obvious cross-correlation, then the prediction error is dependent on the time series, as well as the input. Under these conditions, the model’s general performance significantly decreases and the prediction function will fail. In the NARXNN prediction algorithm, the error autocorrelation graph and the input and error correlation graph were visualized, as shown in Figs. 7 and 8, respectively.

In practice, it is impossible to guarantee complete disparity. When calculating the degree of autocorrelation, the confidence interval should beset to 95%^23^. The error should be maximized when lag is 0, and in all other cases, below the confidence interval. The lower the correlation between the input and the error, the better the results, and the closer its value is to zero in the plot. According to Fig. 7, the autocorrelation coefficient of the temperature prediction error sequence is 10^−3^, it is only maximized when lag is 0, and in other cases, it does not exceed the confidence interval from the third group. These results demonstrate that the temperature prediction error sequence is a random process and does not have any correlation. Figure 8 shows that the input and error correlation is very low, the maximum value of the correlation coefficient’s absolute value does not exceed 0.2, and that all values are within the confidence interval. The combined results from the error autocorrelation analysis and the input and error correlation analysis comprehensively demonstrate that it is feasible to use the model to predict the solar greenhouse temperature data.

### NARXNN prediction model results analysis

To verify the accuracy and adaptability of the model prediction results, the 61 groups of prediction sample sets that were not used to train the model, were verified and analyzed via the heterometric calibration method. Due to the 1:3delay ratio, this process began with the fourth moment; thus, a total of 58 groups were analyzed. Specifically, the sample sets’ measured and predicted values were compared and analyzed, the correlation between these values was calculated, and a correlation analysis plot was generated in which x is the indoor temperature measured value and f is the room temperature predicted value.

**Figure 9:**
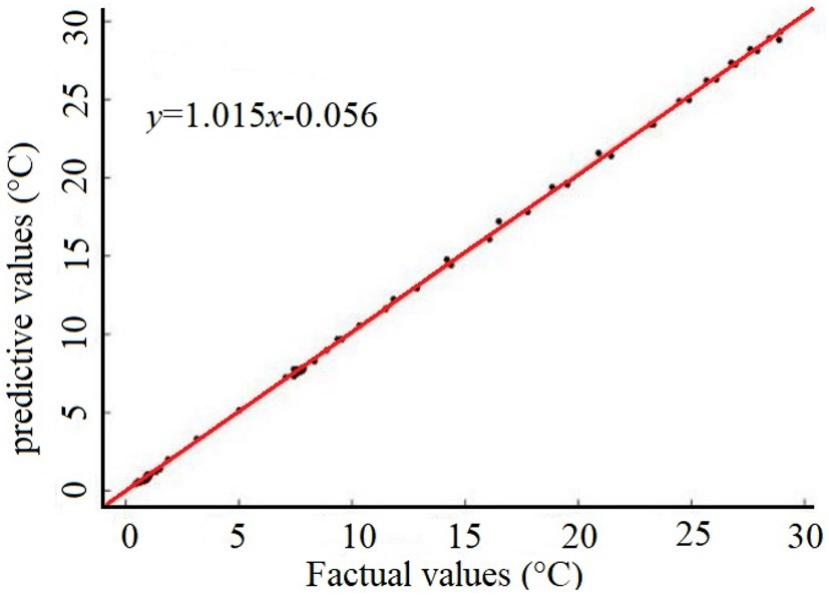
Temperature prediction of NARXNN.

**Figure 10:**
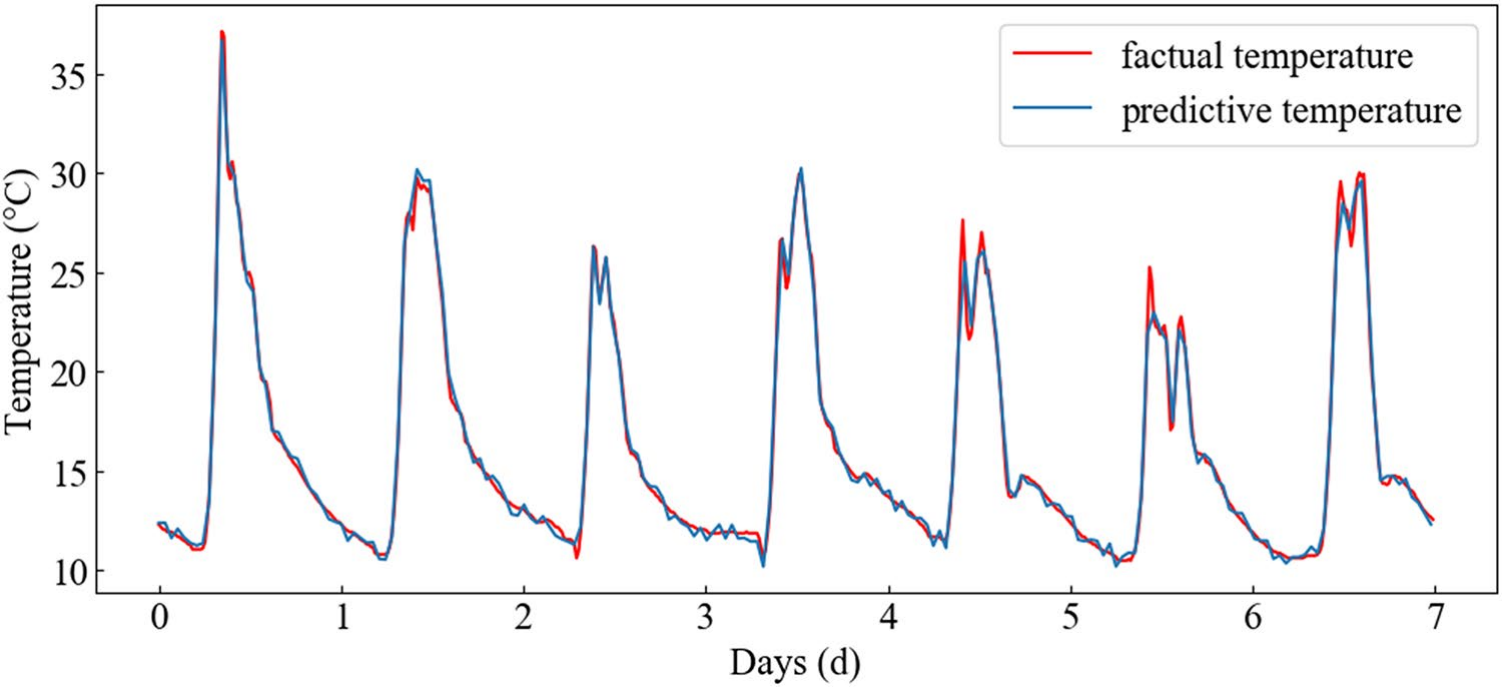
Temperature prediction results.

After fitting a correlation curve between the measured and predicted values (see Fig. 9), it was determined that the coefficient of determination = 0.9976, slope of the correlation curve = 1.015, and the ordinate intercept = − 0.056. These results demonstrate that the measured value is highly correlated with the predicted value. In addition, an error analysis of the prediction results from the same 58 sets was simultaneously performed. Results showed that the maximum absolute error = 0.67 °C. Thus, the solar greenhouse temperature time series prediction model established in this work can achieve high-precision greenhouse temperature prediction. As such, it can successfully be used to provide a scientific basis for forecasting greenhouse conditions, and therefore enable accurate greenhouse temperature regulation. To further display the temperature prediction results, the predicted temperature is displayed in the form of time series as shown in Fig. 10. The trend of predicted results is completely consistent with the real results. The peaks and troughs of daily temperature could be almost perfectly captured. Although there is error in the numerical value, the error is very small and within an acceptable range.

**Figure 11:**
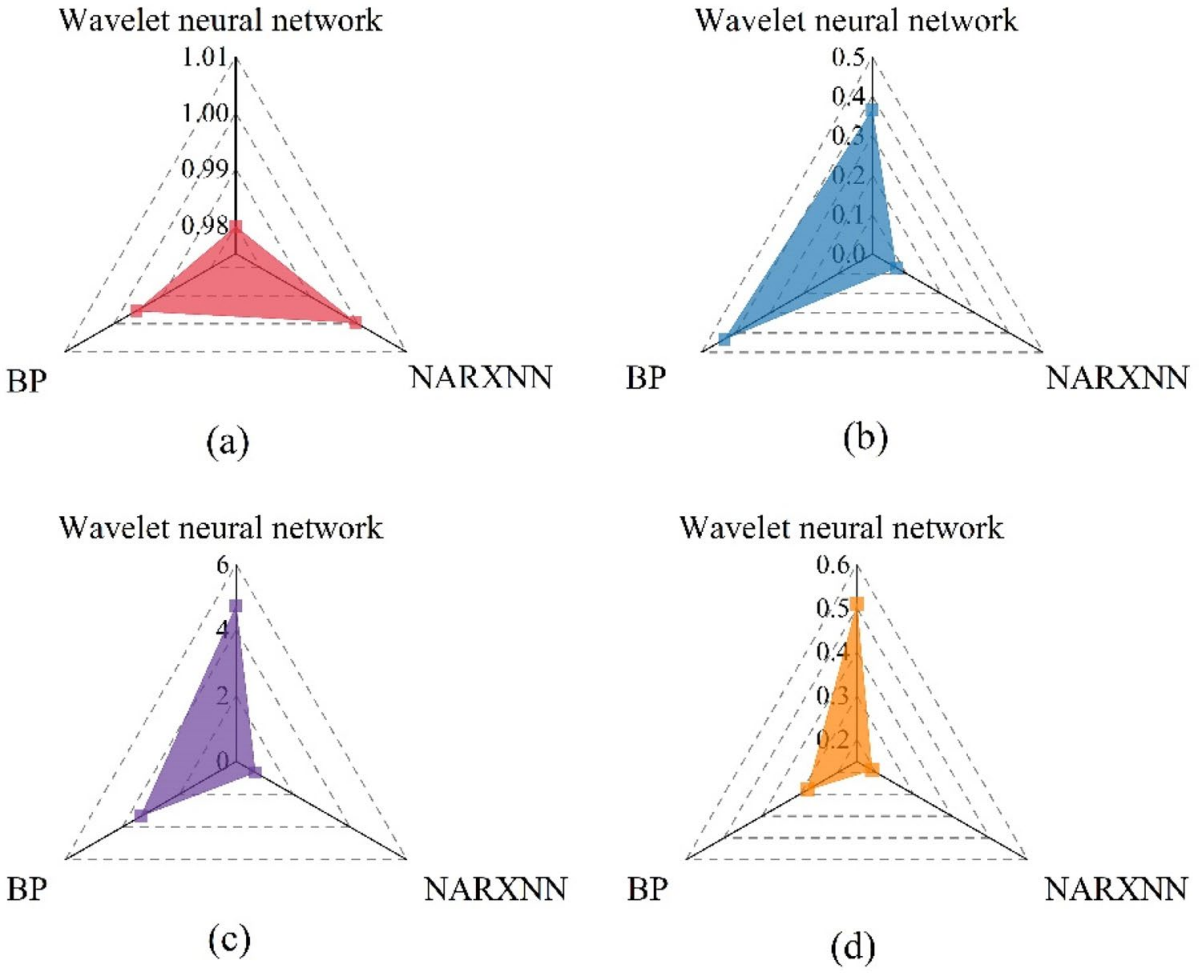
Evaluation index of greenhouse temperature prediction model. (**a**: R^2^, **b**: MSE, **c**: Maximum absolute error, **d**: MAE).

In the research of temperature prediction, neural network algorithm has strong adaptability because of its data-driven characteristics. Wang et al.^24^ adopted BP neural network to obtain a good temperature prediction model for solar greenhouse. Wang^25^ also obtained a temperature prediction model with excellent performance by using the Wavelet neural network. Compared with them, the proposed method adds a temperature feedback mechanism. To further evaluate the performance of the NARXNN based greenhouse temperature prediction model proposed herein, the same training and prediction sets were used in conjunction with the wavelet neural network time series prediction algorithm and the BP neural network to establish a model. Subsequently, the prediction performance of all three algorithms was evaluated. The evaluation results are shown in Fig. 11.

As shown in Fig. 11, the model based on the NARXNN algorithm depicts the best performance, with the R^2^ of 0.9996, the MAE of 0.19, the maximum absolute error of 0.67, and the MSE of 0.12. The wavelet neural network timing analysis prediction algorithm and the BP network both use the historical temperature of the three historical moments as the input and the current moment temperature as the output to train the network. The NARXNN prediction algorithm and the wavelet neural network time series analysis prediction algorithm enhance the sequence learning ability more so than the BP neural network prediction algorithm. As such, when solving the timing prediction problem, the former two algorithms show better prediction performance than the latter. Comparing the wavelet neural network timing analysis prediction with the NARXNN prediction algorithm, the latter adds a delay factor to consider the environmental factor timing characteristics. Subsequently, it uses the main environmental impact factor’s optical radiation value and the indoor and outdoor temperature difference value as the network input to correct the network and predict the temperature at the next moment.

## Conclusions

In this work, the performance of a NARXNN time series prediction model was compared with that of models using the wavelet neural network time series prediction algorithm and BP algorithm. Results showed that the NARXNN time series prediction model depicted both accurate results and the highest performance, and thus, is proposed for solar greenhouse ambient temperature prediction.

Compared with traditional greenhouse temperature prediction models, the model established herein is based on a time series and introduces two main environmental factors as model inputs—temperature difference and light radiation. Moreover, the proposed model depicted a maximum absolute error of 0.67 °C, and model correlation coefficient of 0.9996. Compared with traditional temperature prediction models, the temperature prediction accuracy offered by the NARXNN time series prediction model is greatly improved. Due to the greenhouse environment lag, greenhouse environment temperature regulation needs to be predicted and activated in advance. The model can achieve accurate temperature prediction for the next 20 min, which increases the advance decision-making time, and subsequently offsets the response time of the indoor temperature to the roller shutter quilt action, in effect, enabling proactive control.

The temperature is not only related to the current environmental conditions, but also to the accumulated heat in the past. The proposed model does not introduce accumulated heat, which limits the long-term temperature prediction performance. In the future research, the parameters related to heat accumulation, such as light duration, can be used as model inputs.

## Data Availability

The datasets used and/or analyzed during the current study available from the corresponding author on reasonable request.
